# Some Remarks on Hernia

**Published:** 1908-09-26

**Authors:** 


### Surgery.

### SOME REMARKS ON HERNIA.

### I. INGUINAL HERNIA.

This is one of the commonest of all surgical maladies. An investigation of the records of any London hospital will reveal the fact that, with the single exception of appendicitis, the number of admissions for the treatment of hernia exceeds that of any other single disease for a given period. Of course, men of the hospital class have as a rule laborious employments, which necessitate a heavy strain; but that this is not the only factor is demonstrated by the fact that the preponderance of hernia is almost as great among the well-to-do as it is among the labouring classes.

Much has been written about the causation of inguinal hernia, and it is customary to divide cases of inguinal hernia into two classes—(*a*) congenital and (*b*) acquired. Among the congenital causes of hernia may be mentioned non-obliteration of the funicular process, the possession of an abnormally long mesentery or omentum, and lastly phimosis.

The acquired form is said to be due to any one of a large number of causes, all of which act in the same way—namely, by producing an increase of the normal intra-abdominal pressure. Violent exercise and laborious employments, particularly those which demand the lifting of heavy weights, act in this way; and so do chronic bronchitis and constipation.

Now it is usually taught that the second class is the most important, and that the great majority of herniæ coming on in adult life are acquired. So, indeed, in a sense they are. But the writer is inclined to think that whenever a hernia is acquired, some predisposing condition was previously present. This cannot be denied in those cases in which the sac is found to be composed of the completely unobliterated funicular process and the hernial contents are in direct contact with the upper pole of the testis. It is surely not too much to argue that some predisposing condition is present in all cases.

For let us suppose that in a completely normal individual there is a sudden increase of the intra-abdominal pressure. If the anatomy of his inguinal canal is perfect, this increase will only tend to approximate the anterior and posterior walls of the canal and so increase its valve-like action in preventing the escape of abdominal contents. And it is hard to imagine that any single effort, as in lifting a weight, or series of efforts, as in bronchitis, should have sufficient power not only to derange the normal anatomy of the inguinal canal, but also to push down into it a process of peritoneum. If, on the other hand, we suppose that a peritoneal diverticulum, due to some congenital abnormality, already occupies the site of the inguinal canal, a very slight increase of intra-abdominal pressure will force a coil of intestine into the mouth of the preformed sac, with the result that it will act in a manner somewhat similar to that of a hydrostatic dilator, progressive coils of intestine being pushed on into the sac until a fully formed hernia develops.

In support of this argument, it may be pointed out that this is exactly what does happen in the true cases of congenital inguinal hernia. The great majority of them do not develop until adult life is reached. During the years of adolescence the walls of the unobliterated funicular process are in apposition. Then some strain starts the process, and a hernia is developed, which is thus quite as much an acquired as a congenital condition.

There are, however, cases to which the argument cannot apply: these are the so-called direct inguinal herniæ. In these, as the result of a violent effort or strain, a hernial protrusion presents itself in the inguinal region, bursting its way through the layers of the abdominal wall in this situation quite irrespective of the anatomical conformation of the part. But these cases are excessively rare; in fact, the writer is not quite convinced that he has ever seen a genuine case of direct inguinal hernia.

If this hypothesis is true—that in nearly all cases there is a preformed peritoneal sac, the obvious corollary is that the essential point in the operation for radical cure is the removal of the sac: in comparison with this, the method employed in the other steps of the operation is a matter of minor importance. If one takes the trouble to trace the after history of cases which have been operated on for inguinal hernia, one finds that the percentage of successful cases—that is to say, cases in which there has been no recurrence of the hernia five years after the operation—is a very high one; and, further than this, that it seems to make no difference what operation was originally performed. A further inference may also be drawn—that truss treatment cannot be regarded in any case as a means of effecting a permanent cure. Of course, in an adult a truss is given to keep the hernia in position mechanically in cases in which an operation has been refused or is contra-indicated on general grounds. But it is quite commonly stated that truss pressure applied in quite young patients may cure the hernia. This cannot be the case. The most it can do is to cause the walls of the sac to adhere to each other, and so temporarily to close it. Even if this object is achieved the patient possesses an obliterated hernial sac whose walls may subsequently be forced apart by the slightest increase of intra-abdominal pressure, with the result that a second hernia develops.

Proof of this statement that truss pressure is an inadequate form of treatment for hernia is afforded by the progressively decreasing number of cases which are treated by mechanical appliances and by the increase in the number of cases which are annually subjected to operation. And it is not only an inadequate, but it is also a very irksome form of treatment. In some varieties of hernia it is almost impossible, with all the skill in the world, to apply a properly fitting truss; and, finally, the truss has yet to be invented which will enable a patient with a hernia to pursue a laborious occupation without incurring considerable risk.

